# Excipient for External Application

**Published:** 1890-03-29

**Authors:** 


### EXCIPIENT FOR EXTERNAL APPLICATION.

In the matter of local applications to the skin, the Germans are far ahead of our practitioners and pharmacists, most of our ointments being, to say the least, nasty, and if not rubbed in, very soon rubbed off. Unna's mulls, however, are difficult of preparation, and can seldom be had here, but as a vehicle for powders, &c., nothing can be more simple, cleanly or convenient, than a mixture of glycerine and water in equal parts, with 5 per cent. or a little more of gelatine. It will, when liquified by warmth, take up any drug, and forms a clean and firmly adherent, though perfectly flexible coating on the surface.

